# Impact of physical function on indeterminable anaerobic threshold in patients with heart failure

**DOI:** 10.20407/fmj.2020-015

**Published:** 2020-10-10

**Authors:** Sayano Ueda, Yuji Kono, Ryo Yamada, Tomoya Ishiguro, Masataka Yoshinaga, Satoshi Okumura, Wakaya Fujiwara, Mutsuharu Hayashi, Yoichiro Aoyagi, Eiichi Saitoh, Yohei Otaka, Hideo Izawa

**Affiliations:** 1 Department of Cardiology, School of Medicine, Fujita Health University, Toyoake, Aichi, Japan; 2 Department of Rehabilitation, Fujita Health University Hospital, Toyoake, Aichi, Japan; 3 Department of Rehabilitation Medicine I, School of Medicine, Fujita Health University, Toyoake, Aichi, Japan

**Keywords:** Anaerobic threshold, Cardiopulmonary exercise testing, Cardiac rehabilitation, Exercise tolerance, Heart failure

## Abstract

**Background:**

Anaerobic threshold (AT) during cardiopulmonary exercise testing (CPET) is not always determinable in patients with heart failure (HF). However, little is known about the clinical features of patients with HF who have indeterminable AT. Therefore, the present study aimed to clarify the clinical features of such patients.

**Methods:**

A total of 70 patients with HF (58 males; age: 68±12 years) who underwent CPET during hospitalization were divided into two groups: determinable AT (*n*=50) and indeterminable AT (*n*=20). Physical function, echocardiographic results, and laboratory findings were subsequently determined.

**Results:**

Univariate analyses showed that the indeterminable AT group had significantly higher age and left ventricular ejection fraction, and significantly lower body mass index, calf circumference, handgrip strength, walking speed, serum hemoglobin, and serum albumin than the determinable AT group. Multiple logistic regression analysis identified handgrip strength and walking speed as independent predictive factors for indeterminable AT. Receiver-operating characteristic analyses revealed that handgrip strength of 21.2 kg and walking speed of 0.97 m/s were optimal cutoff values for differentiating patients who were likely to experience indeterminable AT.

**Conclusions:**

The present study identified handgrip strength and walking speed as powerful predictors for indeterminable AT with HF.

## Introduction

Cardiopulmonary exercise testing (CPET) is a well-accepted method for evaluating exercise tolerance in patients with heart failure (HF) because it can provide useful information, such as disease severity and pathophysiological condition, and is a prognostic predictor.^1–4^ Anaerobic threshold (AT) is widely used as an index of exercise tolerance, primarily because it does not require maximal exercise for its determination.^5^ AT has also been recommended as an indicator of optimal exercise training intensity during cardiac rehabilitation.^6^ However, AT cannot always be determined in patients with HF. A previous study suggested that indeterminable AT may be associated with poor prognosis in patients with HF.^7^ However, little is known about the clinical features of patients with HF who have indeterminable AT.

Sarcopenia is generally accepted as a geriatric syndrome that entails progressive loss of skeletal muscle mass and lower physical function. In a previous study, patients with HF were shown to have increased prevalence of sarcopenia, which is closely associated with increased risk of clinical events, including poor prognosis.^8^

Based on these findings, we hypothesized that lower physical function, such as that occurring during sarcopenia, is closely related to indeterminable AT. Thus, the present study aimed to clarify the clinical features of patients with HF who have indeterminable AT.

## Methods

### Study subjects

This was a noninterventional study that used already existing data collected for clinical purposes. According to the ethical guidelines for medical and health research involving human subjects in Japan, we made an announcement for the population to which the study subjects belonged, with respect to the detail of the study, and provided an opportunity for refusal or withdrawal. The study was approved by the Fujita Health University Ethical Review Board (HM 17-104).

The study design was cross-sectional, and the eligible participants were 228 patients admitted to Fujita Health University Bantane Hospital for worsening HF between April 2016 to March 2019. Patients who could not walk 10 m independently (*n*=97), had severe dementia (*n*=10), had a history of severe obstructive lung disease (*n*=6), died during admission (*n*=12), or did not perform CPET at discharge (*n*=33) were excluded. Accordingly, 50 patients whose AT could be determined (determinable AT group) and 20 whose AT could not be determined (indeterminable AT group) during CPET were evaluated in the present study. Clinical data, including laboratory measurements and echocardiography results, and physical function measurements, including handgrip strength, calf circumference, and walking speed, at 1 week before discharge were obtained from clinical charts.

### Exercise testing

Each patient underwent CPET on a cycle ergometer at a progressively increasing work rate until they reached maximum tolerance. The test protocol was in accordance with the recommendations of the American College of Sports Medicine (ACSM).^9^ All patients began at 10 W for a 3-min warm-up, followed by a 10 W/min ramp increment protocol up to the termination criteria.^10^ The test termination criteria were based on the ACSM criteria. A qualified exercise physiologist conducted each test with physician supervision. Continuous 12-lead electrocardiogram monitoring was employed, while blood pressure was measured every minute during exercise and throughout the recovery period. Respiratory gas exchange variables, including oxygen uptake (VO_2_), carbon dioxide production (VCO_2_), and minute ventilation (VE), were acquired continuously throughout the exercise testing using the Aero Monitor AE-301 (Minato Medical Science, Osaka, Japan) through which gas exchange data were obtained with each breath. AT was determined using several methods based on conventional criteria: the point at which the plot of VCO_2_ against VO_2_ first departed from linearity during CPET (V-slope method), the point at which VE/VO_2_ increased after being stable or decreased while VE/VCO_2_ remained constant or was decreasing, and the point at which the gas exchange ratio began to increase more steeply after being stable or slowly rising.^11–14^ In the absence of clinical events, CPET was self-interrupted by the patients stating that they had reached maximal effort. All CPET procedures were performed by a cardiologist and a physical therapist who specialized in CPET.

### Physical function

Physical function was evaluated based on handgrip strength, calf circumference, and walking speed. Handgrip strength was measured three times for each hand using a Jamar dynamometer, with the highest value selected for analysis. For the measurements, participants were asked to sit with their wrist in a neutral position and their elbow flexed at 90°. Calf circumference was measured to the nearest 0.1 cm in the prone position using a non-elastic tape measure and was recorded as the average of two trials for each leg that were subsequently combined to obtain an average for both legs. For the measurements, the tape measure was placed around the calf without compressing the subcutaneous tissue and was moved along the length of the calf to obtain the maximal circumference. Walking speed was evaluated using the 10-m usual-pace walk test wherein subjects were requested to walk at a comfortable pace for 14 m, and the first 10 m was timed. The test was conducted twice and the fastest speed was selected for analysis.

### Statistical analysis

Data are presented as mean±standard deviation for continuous variables and as percentage for categorical data. Differences between the two groups were evaluated by Student’s unpaired *t*-test or the Mann–Whitney U test for continuous variables and by the chi-square test or Fisher’s exact test for categorical variables. Variables with values of *p*<0.1 on bivariate analysis were entered into multiple logistic regression analysis using a forced entry method to determine independent predictors for determinable AT. Receiver-operating characteristic (ROC) curves were constructed, and the area under each curve was evaluated to select a cutoff value for predicting determinable AT. All analyses were performed using the SPSS 21.0 software package (SPSS Inc., Tokyo, Japan) with values of *p*<0.05 were considered statistically significant.

## Results

### Clinical characteristics

A total of 70 patients (56 men) were enrolled, among whom 20 (28.5%) belonged to the indeterminable AT group and 50 belonged to the determinable AT group. The baseline clinical characteristics are presented in Table 1. The mean age was 68±12 years (range: 40–94 years), with the indeterminable AT group having older age than the determinable AT group. The indeterminable AT group had lower male prevalence and body mass index than the determinable AT group. No differences in use of angiotensin-converting enzyme inhibitors (ACEi), angiotensin II receptor blockers (ARBs), beta-blockers, and diuretics were observed between the two groups. No significant difference in N-terminal pro brain natriuretic peptide (NT-proBNP) was observed between the two groups. The indeterminable AT group had higher left ventricular ejection fraction (EF) based on the Simpson method, lower handgrip strength, calf circumference, and walking speed, and lower respiratory exchange ratio (RER) and peak VO_2_ compared with the determinable AT group.

### Multiple logistic regression analysis

Table 2 shows the results of the multiple logistic regression analysis for predictors of determinable AT. Handgrip strength and walking speed were identified as independent predictors for determinable AT, even after adjustment for confounding factors.

### ROC analysis

**Figure 1:**
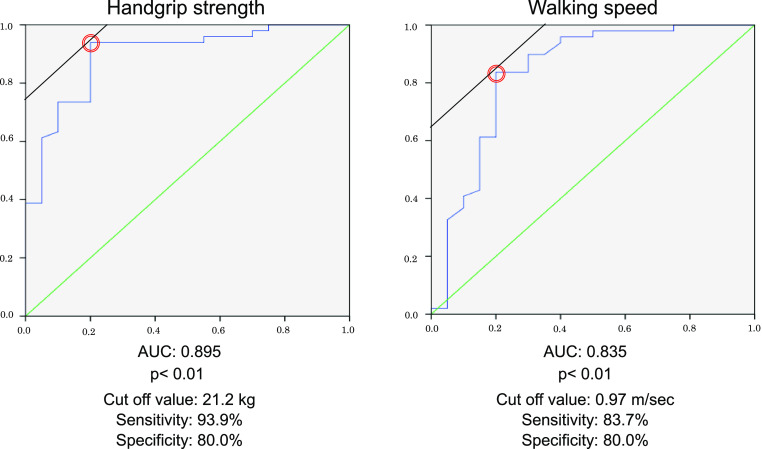
Receiver-operating characteristic curves of handgrip strength and walking speed for predicting the presence of AT.

Figure 1 shows the ROC curves of handgrip strength and walking speed for predicting determinable AT. Handgrip strength and walking speed had an area under the curve of 0.895 (95% confidence interval [CI]: 0.81–0.97; *p*<0.01) and 0.835 (95% CI: 0.71–0.95; *p*<0.01), respectively. A cutoff value of 21.2 kg for handgrip strength yielded a sensitivity of 93.9% and a specificity of 80.0%. Similarly, a cutoff value of 0.97 m/s for walking speed yielded a sensitivity of 83.7% and a specificity of 80.0%.

## Discussion

The principal finding of the present study on patients with HF was that the indeterminable AT group had lower physical function than the determinable AT group. To the best of our knowledge, this is the first report to demonstrate that lower physical function could be closely related to indeterminable AT during CPET.

Our findings showed that approximately 20% of patients with HF had indeterminable AT. Moreover, the ROC analyses showed that cutoff values of 21.2 kg for handgrip strength and 0.97 m/s for walking speed were able to predict indeterminable AT. Although physical function was strongly associated with indeterminable AT in the present study, left ventricular EF and NT-proBNP were not.

AT is the exercise level above which anaerobic metabolism is added to aerobic metabolism. AT is determined on the basis of oxygen delivery and oxygen extraction/utilization in the skeletal muscles.^15–17^ Oxygen delivery is influenced by cardiac output, blood flow distribution, systematic vascular resistance, endothelial function, and arterial oxygen content.^18–20^ Oxygen extraction/utilization is influenced by muscle mass and muscle quality, including muscle fiber type, mitochondrial structure and function, and activation of enzymes associated with energy metabolism.^15,21–23^ Several physiological mechanisms may explain why AT is indeterminable in some patients, the most likely being uneven intramuscular and intermuscular blood flow distribution during exercise, uneven oxygen flow resistance between the capillary bed and mitochondria, and the presence of muscular fibers with uneven O_2_ extraction/utilization. Briefly, the time frame during which anaerobiosis develops within different muscle fibers in a ramp protocol exercise can become considerably wide, meaning that a threshold shared by the majority of muscle fibers no longer exists.^7^ As described above, muscle impairment could be considered a mechanism for indeterminable AT among patients with HF who have low physical function, as shown in this study. Another underlying mechanism for indeterminable AT could be insufficient workload during CPET. CPET can be only considered representative of maximal effort when RER >1.05 has been reached,^24^ given that RER >1.05 at peak effort implies usage of anaerobic metabolism to produce adenosine triphosphate regardless of AT identification.^25^ Indeed, some patients in the indeterminable AT group did not reach RER >1.05. Therefore, such patients may be considered to have completed CPET before reaching AT because of low physical function.

While the present study had an indeterminable AT prevalence rate of 20%, a previous study that evaluated only patients with reduced EF had an indeterminable AT prevalence rate of 9.4%.^7^ Our study included patients with both reduced and preserved EF. Notably, patients with HF who have preserved EF generally have characteristics of older age or female sex with frequent low physical function. Thus, inclusion of patients with preserved EF may be a reason for the higher indeterminable AT prevalence rate observed in the present study compared with the previous study.

Our ROC analyses for predicting the presence of AT revealed cutoff values of 21.2 kg for handgrip strength and 0.97 m/s for walking speed. These cutoff values were almost equal to those in the simplified Japanese criteria for sarcopenia,^26^ which were handgrip strength of <20 kg for females and walking speed of <1.0 m/s. Sarcopenia has been associated with not only loss of muscle mass, but also muscle dysfunction and impaired physical performance. Changes in muscle fiber distribution, blood flow, mitochondrial structure and function, and oxidative stress affect muscle loss and muscle quality impairment.^27^ Considering that the muscle loss and muscle dysfunction observed in sarcopenia correspond to the physiological features of patients with indeterminable AT, the results of the present study suggest that sarcopenia may be a potential determinant for indeterminable AT.

CPET is commonly used in clinical practice for several purposes, including evaluation of exercise tolerance, HF severity, etiology of symptoms, and exercise training intensity.^4^ However, in patients with HF who have indeterminable AT, CPET may not be useful for deciding exercise training intensity and may provide only limited information. Therefore, the purpose of CPET among patients with presumed sarcopenia should be clarified, and different assessment methods such as a field walking test should be utilized to evaluate exercise tolerance or exercise training intensity. Unfortunately, we did not conduct a follow-up study and it is unclear whether the patients with indeterminable AT had a poor prognosis. Large-scale prospective studies are needed to address this issue.

There are several limitations to the present study. First, the study was performed in a small number of patients, which could possibly limit the interpretation of our results. Second, the patients had a low ACEi/ARB prescription rate of about 50%. A previous large-scale registry study reported that patients with older age and HF with preserved EF were less likely to receive ACEi/ARB therapy.^28,29^ These may be the reasons why our patients had a low prescription rate of ACEi/ARB. However, there was no difference in the prescription rate of ACEi/ARB between the two groups. Accordingly, the low ACEi/ARB prescription rate did not influence the results. Third, a previous study reported the presence of interobserver variability in determination of AT.^30^ However, the expiratory gas and ventilation data were recorded and analyzed by two CPET experts who were blinded to the findings in the present study.

In conclusion, the present study revealed that indeterminable AT was strongly related to low physical function in patients with HF. Therefore, the results suggest that the presence of sarcopenia should be evaluated prior to CPET, and that CPET does not constitute an appropriate exercise prescription for patients with HF who have low physical function.

## Figures and Tables

**Table1:** Baseline clinical characteristics

	Determinable AT group (*n*=50)	Indeterminable AT group (*n*=20)	*p*
Age, years	65±11	76±12	<0.01
Male sex, *n* (%)	47 (94)	12 (60)	<0.01
BMI, kg/m^2^	24.0±5.1	21.6±3.8	0.04
Cause of heart failure, *n* (%)			<0.01
CAD	16 (32)	7 (35)	
DCM	28 (56)	3 (15)	
Others	6 (12)	10 (50)	
Atrial fibrillation, *n* (%)	9 (18)	6 (30)	0.27
Pharmacotherapy, *n* (%)			
ACEi/ARB	28 (56)	9 (45)	0.41
Beta-blocker	34 (68)	15 (75)	0.56
Diuretics	34 (68)	14 (70)	0.87
LVEF, %	43.4±14.1	54.2±17.2	<0.01
HFrEF/HFpEF, *n*	21/29	4/16	0.08
Serum hemoglobin, g/dL	14.0±2.4	12.2±2.4	<0.01
Serum albumin, g/dL	3.9±0.4	3.6±0.4	0.10
eGFR, mL/min/1.73 m^2^	60.9±18.0	59.5±22.2	0.79
NT-proBNP, pg/mL	2516±3962	4950±7554	0.08
Calf circumference, cm	35.1±4.3	31.5±3.6	<0.01
Handgrip strength, kg	31.7±6.9	19.6±6.5	<0.01
Walking speed, m/s	1.26±0.29	0.88±0.35	<0.01
Peak VO_2_/W, mL/kg/min	15.5±3.9	10.8±2.8	<0.01
Peak RER	1.16±0.09	1.07±0.09	<0.01
EOV, *n* (%)	6 (8)	4 (5)	0.38

Data are shown as mean±SD, unless otherwise indicated. AT, anaerobic threshold; BMI, body mass index; CAD, coronary artery disease; DCM, dilated cardiomyopathy; ACEi, angiotensin-converting enzyme inhibitor; ARB, angiotensin II receptor blocker; LVEF, left ventricular ejection fraction; HFrEF, heart failure with reduced ejection fraction; HFpEF, heart failure with preserved ejection fraction; eGFR, estimated glomerular filtration rate; NT-proBNP, N-terminal pro brain natriuretic peptide; Peak VO_2_, peak oxygen uptake; Peak RER, peak respiratory exchange ratio; EOV, exercise oscillatory ventilation.

**Table2:** Multiple regression analysis for predictors of determinable AT

	B	SE	Wald	*p*	Exp (B)	95% CI of Exp (B)	
						Lower	Upper
Age	−0.012	0.050	0.060	0.807	0.988	0.896	1.089
BMI	0.016	0.251	0.004	0.951	1.016	0.621	1.661
Male	0.495	1.084	0.209	0.648	0.609	0.073	5.104
Handgrip strength	0.311	0.115	7.314	0.007	1.365	1.090	1.711
Walking speed	0.512	0.219	5.457	0.019	1.699	1.085	2.557
Calf circumference	0.016	0.285	0.003	0.954	0.984	0.563	1.720
Serum hemoglobin	0.031	0.251	0.015	0.902	0.969	0.593	1.586
LVEF	−1.815	1.277	2.019	0.155	6.140	0.502	75.038

BMI, body mass index; LVEF, left ventricular ejection fraction; CI, confidence interval.
